# Supplementary material for: Phenotypic and genotypic detection of extended spectrum beta lactamase enzyme in Klebsiella pneumoniae
Source: PLoS One. 2022 Sep 29;17(9):e0267221. doi: 10.1371/journal.pone.0267221 (PMC9522270; doi:10.1371/journal.pone.0267221)
Supplement: Attachment — Submitted filename: manuscript-26-10-2021.docx [file pone.0267221.s002.docx]

| N0 | **Line number** | **Reviewer’s Comments/Suggestions** |
| --- | --- | --- |
| 1 | Abstract | Most results of antibiotic not clarify clearly in abstract section |
|  | Keywords | Need to arrange alphbetically |
| 2 |  | The manuscript contains many grammatical errors that must be corrected especialy the discussion and introduction section need too much correction |
| 3 | highlighted in Green | Correct the word |
| 4 | highlighted in Red | Delete word or character |
| 5 |  | Many Word not writed in fully form like” EBLS,ICU,U.S.A,MICs,OPD)and others, also the term EBLS sometimes writed “ESBLS” other palces writed “ESBLs” it must write in unique and scinetific style |
| 6 | Highlighted in Yellow | Give a space |
| 7 | Results | - İn this sectio the concentration of each antibiotiv must mentioned inside the (table 4).  - (P-value with 0.00001) this wrong value (correct the value). |
| 8 | Discussion | The authors mentioned in this section this wrong reference lined with yellow” The other drug such as amoxicillin-clavulanic acid also shows high resistance rate (96.29%), the same result was reported in Erbil , Najaf and Duhok / Iraq (Gupta etal.(2003), (Aljanaby etal.(2018), (Qadir (2019). Respectively. So Gupta and his collegous study founded in U.S.A |
| 9 | References | All the refernce not in one unique style; the year of publication sometime after the name of authors and sometimes after the title of article, sometiems after the year of publication come comma others semicolon |
| 10 | Others important notice | - Many word wtrited wrongly like „standardise,Fifty four,30 µg must become  30µg (without any space),TEM and SHV writed with different format like balSHV,blasCTM  - Table 1,2 need to re-type in word format not in picture foramt  - Chi-sequare and correlation analysis test mentioned in statistical anlalysis section but actualy not calculate for the data (for comparision), the authors must delete these characters , also the authors must mentioned which version of M.soft excel they used  - Table 3, 4,5 ,6 and 7 must re-type again with word format adn without coluration .  - The manuscript not contanin conclusion and any Acknowledgement. |

**Phenotypic and genotypic detection of extended spectrum beta lactamase enzyme in *Klebsiella Pneumoniae***

Authors :

1. Aso Bakr Mohammed / High Diploma / Bs.c. biology / Shar Teaching Hospital / Microbiology department / Sulaimani / Iraq .

[asobiology@gmail.com](mailto:asobiology@gmail.com)	Mobile Number : 07701910764

2.Dr. khanda Abdullateef Anoar Assistant professor / MBChB , PH.D Clinical Microbiology / Head of Microbiology Department / College of Medicine /University of Sulaimani / Iraq

[Khanda.anwar@univsul.edu.iq](mailto:Khanda.anwar@univsul.edu.iq) Mobile Number 07707751600

[khandaanwar@yahoo.com](mailto:khandaanwar@yahoo.com)

Abstract

**Background** : *Klebsiella* species are ubiquitous in nature and can be found in the natural environment and on mucosal surfaces of mammals and it is an important multidrug-resistant (MDR) pathogen affecting humans and is a major source for hospital acquired infections.

The aim of this study is to investigate the prevalence of ESBL among *Klebsiella Pneumoniae* isolates by phenotypic methods from different hospital wards and detection of ESBL resistance genes such as TEM and SHV in Sulaimani city/ Kurdistan– Iraq.

**Methods** : *Klebsiella Pneumoniae* isolates were collected from different clinical samples from different hospitals , the isolates were identified by standard technique. Screening of ESBLs was undertaken by using double disk diffusion and standard disk diffusion methods. Real-time PCR was used for genotypic detection of TEM and SHV genes according to the standard protocol.

**Result** : Out of 54 *Klebsiella pneumoniae* isolates; 28 were ESBL positive, The pattern of antimicrobial susceptibility showed resistants to most of antimicrobial agents .Both TEM and SHV genes were detected among 28 ESBL positive (51.85%) of *Klebsiella pneumoniae* isolates by using Real-time PCR method.

**Conclusion**; SHV gene was detected in most of the isolates of ESBL producers of Klebsiella Pneumoniae.

**Key words :** multidrug- drug resistant, Extended spectrum beta lactamase , HSV gene , TEM gene

**Introduction**

Extended-spectrum beta-lactamase (ESBL)-producing bacteria is plasmid-mediated enzymes capable of hydrolyzing a β-lactam bond in commonly prescribed β-lactam antibiotics, such as penicillins, broad spectrum cephalosporins and aztreonam (Monald..*etal* 2008), these enzymes have been clustered into nine different structural and evolutionary families based on their amino acid sequence TEM and sulphydryl variable SHV were the major types. However, CTX-M type is more common in some countries (Gupta ..*etal* 2003 ) and they are rapidly evolving and produced by Gram negative bacteria, and have the ability to hydrolyse all cephalosporins, aztreonam, and related oxyimino-beta lactams as well as older penicillins but this can be inhibited by clavulanic acid (Elnagar..*etal* 2010).

The plasmids that harbor ESBL genes also carry genes for other antibiotic classes such as aminoglycosides, chloramphenicols, sulfonamides, trimethoprims, and tetracyclines. Thus, Gram negative bacilli containing these plasmids mostly are MDR bacteria (Rozwandwicz…*etal* 2018).

A large number of outbreaks of infections due to ESBL producing organisms have been described all over the world. In some hospitals, initial outbreaks of infections have been supplanted by endemicity of the ESBL producing strains (Paterson & Bonoma 2005 ). This may lead to increased patient mortality, therefore, control of the initial outbreak of ESBL producing organisms in a hospital or specialized unit of a hospital is of critical importance (Pishtiwan & khadijia 2019).

About 400 different types of ESBLs have been recognized around the world, among which TEM and SHV were more prevalent, mutations occurring in the genes encoding TEM and SHV enzymes lead to the development of ESBLs that have an expanded substrate profile (Latipour & Dmamavandi 2016).

Both TEM and SHV types are derived from penicillinase that are characterized by several single amino-acid substitutions, these mutations allow them to hydrolyze extended spectrum cephalosporins (Mohammed & Abass 2019 ).

TEM-1 is the most commonly encountered β-lactamase in Gram-negative bacteria. Up to 90% of ampicillin resistance in *E. coli* is due to the production of this enzyme, and it is also responsible for the ampicillin and penicillin resistance that is seen in *H. influenzae* and *N. gonorrhoeae* (Mawlood ..*etal* 2008 ) .

The fact that patients most likely to become infected with ESBL-producing Enterobacteriaceae are those with prolonged stays in the ICU. Failure to rapidly detect ESBL-producing strains may result in serious consequences, such as treatment failure and death (Navon-venza etal.( 2017).

It is estimated that Klebsiella species cause 8% of all hospital-acquired infections. In the USA it comprise 3% to 7% of all nosocomial bacterial infections, this place them among the eight most common pathogens in hospitals and second only to *E. coli* as the most common cause of Gram-negative sepsis (Jean & hsueh 2011), while urinary tract is the most common site for infection of *Klebsiella* species and it accounts for 6 to 17% of all nosocomial urinary tract infections (UTI) and shows an even higher incidence in specific groups of patients at risk, such as patients with neuropathic bladders or with diabetes mellitus (Tsai..*etal* 2010).

The likelihood of *Klebsiella pneumoniae* hospital-acquired infections is greatly increased by the presence of invasive devices, such as catheters, and ventilator associated pneumoniae in hospitalized patients (Afroz etal.(2017).

Antimicrobial resistance in *Klebsiella Pneumoniae* has been attributed largely to the acquisition of large, self-conjugating plasmids. These are extra chromosomal DNA molecules that encode factors promoting the initiation of replication, independently from the replication of the bacterial chromosome, maintain a constant copy number per bacterial cell and regulate their own replication rate (Lue etal.(2014).

Carriage of multiple β-lactamase genes in the same strain is a known ability of *Klebsiella pneumoniae* and may contribute to the selective success of this pathogen (Lee etal.(2016). Combinations of all types of bla genes were reported in this species, this may be due to either carriage of an antibiotic-resistant plasmid encoding an array of antibiotic resistance genes or due to acquisition of transposons containing different bla genes on the same plasmid (Navo- venza etal .(2017).

Many different techniques exist for confirming ESBL production , Phenotypic tests for ESBL detection only confirm whether an ESBL is produced but cannot detect the ESBL subtype. Some ESBLs may fail to reach a level to be detected by disk diffusion tests but result in treatment failure in the infected patient (Schwarz etal.(2010), while those utilizing similar methodology to standardise susceptibility tests are the most convenient for the routine diagnostic laboratory. These all depend on detecting synergy between clavulanic acid and the indicator cephalosporin used in the primary screening (shariif etal.(2017).

Another confirming method involves at least an eight-fold reduction in the MICs of ceftazidime or cefotaxime in the presence of clavulanic acid, The isolates then reported as resistant to all penicillins, cephalosporins and aztreonam (muhamed & Abas 2019).

Early detection of β-lactamase genes was performed using DNA probes those were specific for TEM and SHV enzymes. However, using DNA probes can sometimes be rather labor intensive. The easiest and most common molecular method used to detect the presence of a β-lactamase belonging to a family of enzymes is PCR with oligonucleotide primers that is specific for a β-lactamase gene (Elengar etal.(2010). Nucleotide sequencing remains the standard for determination of the specific β-lactamase gene present and the rare genes also such as *bla*GES and *bla*SFO, or *bla*PER, *bla*TLA and *bla*VEB (philippon etal.(2011).

Molecular methods appears to be sensitive in spite to that its expensive, time consuming and require specialized equipment and expertise (Woodford etal.(2011).

Determination of TEM and SHV genes by molecular techniques in ESBL producing bacteria and their pattern of antimicrobial resistance can supply useful data about their epidemiology and risk factors associated with these infections ( Mlik etal.(2018).

**Methods**

This cross sectional study was carried out in different hospitals (Shar hospital, Teaching hospital, and Dr. Jamal Ahmed Rashid hospital) in Sulaimani city from a period of March to July 2019.Ethical approval was obtained from Directorate of Health of Sulaimani.

Fifty four isolates of *Klebsiella pneumoniae* were collected from all the mentioned hospitals from different infection sites and the samples underwent processing through standard bacteriological technique by doing Gram stain and culture technique for all the isolates (CLSI (2018).

All isolated *Klebsiella* species were tested by string test through formation of a viscous filament ≥5 mm after stretching a bacterial single colony (figure 1) with a standard bacteriological loop on an agar plate (Hagia etal.(2014), shon etal.(2013). . Vitek^®^ 2 biomeireux machine was used for the identification of all isolated *Klebsiella* according to the manufacturer’s recommendations. Bacterial strains which were identified biochemically were preserved for longer period by transferring a fresh colony from newly culture plates to prepare 0.5 mL bacterial suspension in tryptic soya broth and they incubated for 18 hours at 37 ˚C in shaking incubator, after which 0.5 mL of this growth cultures was mixed with 0.5 mL of sterile 80% glycerol solution in a Eppendoruf tube. After labeling the tubes with numbers and dates of preservation and stored in a deep freezer at -70 ℃ (Anoar etal.(2014).

Antibiotic susceptibility pattern of *Klebsiella pneumoniae* isolates were performed according to the Kirby–Bauer disk diffusion method (Bauer etal.(1966) by using 15 commonly used antibiotic agents and all of the inoculated plates were aerobically incubated at 37°C for 18–24 hours, and the results were interpreted based on the instruction provided by the CLSI (2018 ).

**Detection of Extended Spectrum β-lactamase enzyme**

All isolates of *Klebsiella pneumoniae* were screened for ESBL enzyme production by using various antimicrobial disks, ESBL positive meant the bacteria shows high level (co-resistance) to third generation cephalosporin and monobactams such as: Ceftazidime zone ≤ 22 mm, Aztreonam zone ≤ 27 mm, Cefotaxime zone or Ceftriaxone zone ≤ 25 mm (Garrec etal.(2011) .

**Double disk synergy test (DDST)**

The double-disk synergy method is integrated as an adjunct of the routine susceptibility test. In this test, a disk of amoxicillin-clavulanic acid (AMC) 30 µg and disks of the third generation Cephalosporin antibiotic such as Ceftazidime CAZ disk (30 µg), Cefotaxime CTX (30 µg), Ceftriaxone CRO (30 µg), and Cefepime FEP (10 µg), were used together with Aztronam ATM (30 µg),, each disks were placed at a distance of 20 mm apart from the inhibitor disk on a lawn culture of the resistant isolate on Mueller-Hinton Agar plate. The presence of ESBL was inferred when the inhibition zone around any of the antibiotic disks was enhanced on the side of the Clavulanic acid-containing disk, resulting in a characteristically shaped zone referred to as a “keyhole, (figure 2) (Rangachari etal.(2010).

**Detection of ESBL by VITEK 2 System**

Each isolate was tested using the Vitek^®^ 2 system with the antimicrobial susceptibility test extend AST-EXN8 card. This system was designed to perform both screening and confirmatory tests for phenotypic detection of ESBL on the same plate (Batarseh etal.(2015).

**Genotypic detection of ESBL enzyme**

Two genes (*bla*SHV and *bla*TEM) were selected in this study to be analyzed by real time PCR. Bacterial reactivation was performed by thawing frozen samples (bacterial culture and glycerol), and sub cultured on blood agar then incubated for 24 hours, selected colony from blood agar was sub cultured in to Tryptic soy broth for 18 hours incubated at 37 ˚C, and samples were ready for plasmid extraction (Ranjbar etal.(2019).

**Primer preparation**

Two sets of primers were used in this study and the primers were designed by Macrogen/Korea both forward and revers (Table 1), according to what was previously tested (Doosti etal.(2015).

Primers were prepared according to what was recommended by manufacturer (Macrogen) by dissolving each lyophilized primer in 300 µL of free DNA and RNA injection water by using filter pipette tip to prevent contamination, concentration of stock primers was 100 pmol/µL. Then let it to dissolve well and leave it for 15 minute at room temperature and mixed by vortexing, stock primer diluted 1:10 (10 µL of stock primer with 90 µL of DNA and RNA free distil water) to prepare working primer in concentration 10 pmol/µL (Ali& Ismael (2017).

Plasmid extraction was performed as recommended by the manufacturer of (MagCore^®^,RBC bioscience) automated nucleic acid extraction kit (Taiwan), prior to plasmid extraction, the content of the kit was prepared and checked as recommended.

**Table 1 : *bla* SHV and *bla* TEM primer sequence**

**SYBR Green Real-time PCR for *bla*TEM and *bla*SHV detection**

Real-time PCR was used to detect of TEM and SHV genes, PCR was performed by rapid cycling in a reaction volume 20 µL including (master mix, amplification primers, extracted nucleic acid, and DNA and RNA free distil water ). All isolates with ESBL positive by phenotypic methods were underwent plasmid extraction by automated nucleic acid extractor, 7.5 µL of extracted nucleic acid in concentration 10 ng/µL mixed with 10 µL of qPCR master mix and 1.5 of DNA and RNA deionized water, 0.5µl of forward primer and 0.5 µl of reverse primer at concentration 10 ng/µl then added in to the reaction micPCR tube and the condition of PCR, after that the reaction tube subjected the thermo cycling reaction on bio molecular system (BMS) model instrument (Australia) , according PCR condition used in amplification cycle(Table 2) .

**Table 2 : Programs of real-time PCR thermocycling condition of the current study after serial optimization.**

A negative control was included in each run to access specificity of primers and possible contamination. The threshold cycle (Ct) was determined as the PCR cycle at which increased reporter fluorescence above the baseline could be detected.

All samples were run on PCR machine, PCR reaction data were plotted on graphic presentation by Micro software mic PCR program.

Statistical analysis: All data were analyzed by transfer to excel sheet data, Chi square test, fisher exact test, correlation test were applied when possible. *P* value ≤ 0.05 was regarded as significant.

**Results**

Fifty four suspected colonies of *Klebsiella pneumonia* were collected from different hospital laboratories in Sulaimani city, out of 54 samples ; 29 (53.7%) of all specimen were males and 25 (46.3%) female, with male to female ratio 1.15:1.

All colonies of 54 samples showed pink color, moist and mucoid colony on the surface of MacConkey agar and large grey color colony on blood agar with no hemolytic property among all isolated bacteria .Out of the 54 isolated *Klebsiella pneumonia*, only 8 samples were positive to hypermucoviscosity phenomena according to what was described previously by Catalán-Nájera et al.(2017).

Out of 54 specimens which had been received; the highest numbers were taken from ICU 32, (59.26%), followed by dialysis center 5 (9.26%), 3 (5.55%) for each of Pediatric and OPD, and other wards in hospitals 11 (20.37%). The result indicate that among all specimens taken from all hospital wards; urine were 23 (42.6%), followed by Endotracheal aspiration 12 (22.22%), blood 11 (20.37%), catheter tip 4 (7.41), wound swab 3 (5.55%), and 1 (1.85%) was recorded for sputum (Table 3).

All confirmed samples were tested for antimicrobial susceptibility testing, by using Kirby-Bauer disc

diffusion method and the result were analyzed according to Clinical and Laboratory Standard Institute (21).The susceptibility results against 15 antimicrobial agents is shown in the Table 4 and it was found that the most resistant drug were ampicillin followed by amoxiclave and cetftazidim .

**Table 3: Clinical specimens and source of samples of *Klebsiella Pneumoniae***

| Specimen | ICU | Dialysis | Pediatric | OPD | Other | Percentage |
| --- | --- | --- | --- | --- | --- | --- |
| Urine | 9 | 1 | 2 | 3 | 8 | 23 (42.6%) |
| Blood | 8 | 1 | 1 | 0 | 1 | 11 (20.37%) |
| Endotracheal aspiration (ETA) | 11 | 0 | 0 | 0 | 1 | 12 (22.22%) |
| Catheter Tip | 1 | 3 | 0 | 0 | 0 | 4 (7.41%) |
| Sputum | 1 | 0 | 0 | 0 | 0 | 1 (1.85%) |
| Wound swab | 2 | 0 | 0 | 0 | 1 | 3 (5.55%) |
| Total % | 32(59.26%) | 5(9.26%) | 3(5.55%) | 3(5.55%) | 10(20.37%) | 54 (100%) |

**Table 4 Antimicrobial susceptibility testing for *Klebsiella pneumoniae* (n= 54)**

| Antibiotic | R | I | S |
| --- | --- | --- | --- |
| Amc | 54 (100%) | 0 (0%) | 0(0%) |
| AMC | 54(100%) | 0(0%) | 0(0%) |
| PTZ | 35(64.8%) | 3(5.55%) | 16(29.6%) |
| FOX | 26(48.1%) | 1(1.85%) | 27(50%) |
| CAZ | 45(83.33%) | 0(0%) | 9(16.66%) |
| CTX | 41(75.9%) | 0(0%) | 13(24.1%) |
| CPM | 40(74.0%) | 0(0%) | 14(25.9%) |
| ATM | 38(70.37%) | 0(0%) | 16(29.63%) |
| IPM | 20(37.0%) | 0(0%) | 34(62.96%) |
| MEM | 20(37.0%) | 0(0%) | 34(62.96%) |
| AN | 26(48.1%) | 0(0%) | 28(51.9%) |
| CM | 30(55.55%) | 1(1.85%) | 23(42.6%) |
| CIP | 34(62.96%) | 2(3.7%) | 18(33.33%) |
| TMP | 30(55.55%) | 0(0%) | 24(44.44%) |

**Ampicillin (AMP), Amoxicillin-Clavulanic Acid (AMC), Piperacillin-tazobactam (PTZ), Cefoxitin (FOX), Ceftazidime (CAZ), Ceftriaxone (CRO), Cefotaxime (CTX), Cefepime (CFM), Aztronam (ATM), Imipenem (IPM), Meropenem (MEM), Amikacin (AN), Gentamicin (CN), Ciprofloxacin (CIP), Trimethoprim- Sulphomethoxazole (TMP).**

**Extended Spectrum β-Lactamases enzyme detection**

In this study 54 isolates of *Klebsiella pneumoniae* were screened for ESBL production by using standard disk diffusion method (21)?. The result showed that 40 (74.0%) isolates were resistance for 3^rd^ generation of cephalosporins (ESBL producer), while 14 (26.0%) was susceptible for 3^rd^ generation of cephalosporin (non ESBL producer).

All confirmed *Klebsiella pneumoniae* were tested for ESBL by double disk synergy method. The selected bacterial samples were tested against 3^rd^ generation cephalosporin and characteristic key hole appearance were observed in 28 (51.85%) samples . At the same time all samples were tested by Vitek^®^ 2 system for ESBL and the same results were obtained. The rate of both methods was illustrated in Table 5 and statistically this relation is significant (*P* value < 0.05).

**Table 5 ; percentage of ESBL by screen and confirmation test**

**Proportion of multidrug-resistance among isolates of *Klebsiella pneumoniae***

Multidrug resistance pattern were analyzed among all isolated *Klebsiella pneumonia* (Bora etal.(2014), any strain that shows resistant to at least one antibiotic in each group of three or more classes of antimicrobials reported as multidrug-resistance MDR (Ranjbar etal.(2019). Out of 54 samples of isolated *Klebsiella pneumoniae*; 32 (59.29%) were MDR table (6) and among all MDR; 75% were ESBL positive (Table 7) , and statistically this relation is significant (*p* < 0.05).

Table 6: percentage of MDR among *Klebsiella Pneumoniae*

**Table 7: relation of MDR and ESBL**

**Molecular detection of ESBL**

Both ESBL gene (TEM and SHV genes) were selected to be tested in this study by using real-time PCR. The result demonstrated that 26 isolates (92.85%) were positive for *bla*SHV gene (Figure 3& 4) and 15 isolates (53.57%) were positive for *bla*TEM gene (figure 5 ), and 15 isolates (53.57 %) have both *bla*SHV and *bla*TEM genes, and 2 isolates (3.70%) out of ESBL positive isolates found to be negative to both genes.

**Discussion**

*Klebsiella pneumoniae* is one of the most important multidrug resistant pathogenic bacteria that cause several diseases such as, burns infections, urinary and respiratory tract infections, wounds and blood infections and liver abscess (Remya etal.(2019).

All isolated *Klebsiella pneumoniae* in this study were from different wards in hospitals where different samples were analyzed and received from different clinical condition. 42.6% from all samples were urine specimen, this result is in agreement with study done in Erbil-Iraq; whom reporting higher percentage of urine sample 71.7%, this indicate that UTI is the most common infection in hospitals and Klebsiella is the second most common isolated bacteria after *E. coli* in this condition (Ali & Ismael (2017). The likelihood of *Klebsiella pneumoniae* hospital-acquired infections is greatly increased by the presence of invasive devices, such as catheters and ventilator associated pneumoniae in hospitalized patients and risk of infection increase with those patients with neuropathy bladder and diabetes mellitus (Afroz etal.(2017).

The second most common specimen was blood ( 20.37%) , which accounts for more than 70 % were of ICU samples . this indicates the presence of more critical patients in this unite and they need blood culture continuously to exclude sepsis by gram negative bacteria. *Klebsiella pneumoniae* can persist on environmental surfaces and human skin, within the respiratory , and urinary tracts, and easily transferred among patients via clinical operations and examinations, and it has become one of the most frequent outbreaks in intensive care Units (Gue etal.(2017).

Antibiotic profile were tested against all isolated species and it was clear that 100% resistance to ampicillin was recorded which is in agreement to the studies performed in Erbil/ Iraq and Egypt

(Irajian etal.(2017) (Tawgozy & Amin (2018). However; this high level of resistance to ampicillin is not surprising due to constitutive expression of a chromosomally encoded B-lactamase (*bla*SHV-1) which confers resistance to ampicillin, amoxicillin, carbenicillin and ticarcillin (Haeggman etal.(2004).

The other drug such as amoxicillin-clavulanic acid also shows high resistance rate (96.29%), the same result was reported in Erbil , Najaf and Duhok / Iraq (Gupta etal.(2003), (Aljanaby etal.(2018), (Qadir (2019). respectively, and in other countries similar result was reported such as Nigeria, Iran, Saudi Arabia (Ilisyas etal.(2018) (Khosravi etal.(2013), (Alagamy etal.(2009) ,while lower resistance rate were recorded in a study done in Erbil-Kurdistan region-Iraq and Baghdad-Iraq (Pshtiwan & Khadija (2019), (Aziz & Aljuburi (2016) .

Regarding beta-lactam/beta-lactamase inhibitor combinations; the proportion of isolates showing resistance to amoxicillin/clavulanate (96.29%) was significantly higher than resistance to piperacillin/tazobactam (51.85%), the same result was recorded in Kingdom of Saudi Arabia (Hassan etal.(2013) the fact that piperacillin/tazobactam is less commonly used in this locality and in comparison to ampicillin which indicate over used of antibiotic causing selective pressure on bacteria for development of resistance.

The proportions of Klebsiella pneumoniae isolates showing resistance to non-beta-lactam agents such as trimethoprim (57.40%), ciprofloxacin (55.55%), gentamicin (53.70%), and amikacin (40.74%) were recorded, There is a bit difference with the result to what was recorded in a study done in Erbil-Sulaimani-Duhok/ Iraq by Khalid et al (Khalid etal.(2013) , while imipenem and meropenem were the most susceptible antibiotics (63.0 %) against *Klebsiella pneumoniae* isolates followed by Amikacin (59.26%), these results are in agreement with result of studies done in Erbil/Iraq and Iran (Ali & Ismael (2017), (khosravi etal (2013), which stated that imipenem and meropenem were the most effective antibiotics, this result probably due to less frequent use of carbapenem to treat infection in compared to other antibiotics in this country. However, the emergence of resistance to carbapenem requires careful monitoring, as many of the producers are resistant to all antimicrobials posing a serious threat to hospital units, especially among immune compromise patients and among hospitalized patients. This finding was in agreement with data reported from other regional as well as global studies (Ali & Ismael (2017) , (Ilisyas etal.(2018) , (Rudresh & Nagrahthnamma (2011),(Hawsar etal.(2011), which may imply that increased use of carbapenems could potentially further select resistant strains.

The prevalence rate of ESBL was 51.85% among isolated *Klebsiella Pneumoniae* by double disk diffusion method and Vitek^®^ 2 compact system, this result is in consistence with the study carried out in Erbil, Duhokin Iraq and in Iran (Ali & Ismael (2017), (Qadir (2019),(Akbar etal.(2016), while higher rate was reported in Basrah-Iraq (Alghasan etal.(2016) and a lower rate was reported in a study that performed in Iran (Kazemian etal (2019). There are several studies from Turkey, Iran, Spain and Taiwan; recording prevalence rate of ESBL near to what was recorded in this study (Khorsravi (2013) (Bahar (2005) , (Diestra etal. (2004) this difference may be due to sample size type of clinical specimen. This variation in prevalence rate of ESBL may be due to differences in sample size.

The accurate detection of ESBL producing microorganisms is a major clinical problem in the laboratories, requiring not only phenotypic tests but also genotypic tests for all genes associated with β-lactamase production. The prevalence of β-lactamase producers and the distribution of ESBL genotypes are different from one year to another and even vary greatly in different geographical areas (Pitout & Laupland (2008).

ESBL enzyme carries by several genes such as; SHV, TEM which were analyzed in this study, both genes (*bla*SHV and *bla*TEM) were detected by real-time PCR using specific primer sequences which yielded product sizes of 450 bp and 296 bp, respectively.

The result demonstrated that out of 28 ESBL positive strains 26 (92.85%) of them were positive for *bla*SHV while15 (53.57%) of them were positive for *bla*TEM. This means that 92.85% of ESBL producer *Klebsiella pneumoniae* were carried at least one of the both genes; similar result was reported in a study done in Erbil, Sulaimani and Duhok in Iraq by Khalid et al.(Khalid etal.(2013). This result was in agreement with several studies (38,41,53), whom they reported the prevalence of blaSHV gene in Klebsiella pneumoniae isolates near to the prevalence rate in this study but TEM gene were recorded a bit higher rate than this study.

Out of all ESBL positive isolates by phenotypic methods ; 2 isolates showed negative result by genotyping ; this may be due to presence of other ESBL genes such CTX-M, and OXA gene.

Resistance pattern among all isolated *Klebsiella* were analyzed and MDR strain were found in 59.25%; this indicate that not all samples carry MDR phenomenon. The remaining negative isolated may carry other genes such as AmpC, KPC, or other type carbapenemase enzyme but among all ESBL producers 75 % were MDR, similar result were reported in a study in Erbil/ Iraq (Pshitwan & Khadija (2019) and India (Bora etal.(2014). This mostly due to the presence of multiple resistance gene on the same cassette of chromosomal, The plasmids that harbor ESBL genes also carry resistance genes for other antibiotic classes such as aminoglycosides, chloramphenicol, sulfonamides, trimethoprim, and tetracycline. Thus, Gram negative bacilli containing these plasmids mostly are multidrug-resistant bacteria (Rozwandwicz etal.(2018). Multi drug resistance *K. pneumoniae* strains have caused many disease problems worldwide with higher morbidity and mortality rates, posing a considerable threat to public health (Aljanaby etal.(2018).

**Funding and Conflict of interest:**

This research done on our fund without participation of any organization, a part of helping of motioned hospital for allowing us to using their samples after ethical consideration had been taken from directorate of health.

References

Afroz , H., Fakruddin , M., Masud , M. R. & Islam , K. Incidence of and risk factors for hospital acquired infection in a tertiary care hospital of Dhaka, Bangladesh. Bangladesh Journal of Medical Science. 2017. 16, 358-369.

Akbar , D. A., Ali , R. A. & Zahira , K. Screening and prevalence of SHV/CTX-M/TEM Β-lactamase resistant gene in Klebsiella strains isolated bacteria from UTI in preschool age children in Jahroom Iran . 2016; 7, 82-88.

AL-agamy , M. H., Shibl , A. M. & Tawfik , A. F. Prevalence and molecular characterization of extended-spectrum β-lactamase-producing *Klebsiella Pneumoniae* in Riyadh, Saudi Arabia. Annals of Saudi medicine. 2009 ; 29, 253-257.

Alaghasan , Z., Jasim , U. & AL-mussawi , A. A. Extended Spectrum β-lactamase (ESBL)-producing *Klebsiella Pneumoniae* isolated from Burn Wound Infections. Int. J. Curr. Microbiol. App. Sci. 2016 5, 400-404.

Ali , F. A. & Ismael , R. M. Dissemination of *Klebsiella pneumonia* and *Klebsiella oxytoca* Harboring bla TEM genes isolated from different clinical samples in Erbil City. Diyala Journal of Medicine. 2017; 12, 40-51.

Aljanaby A., Tuwaij N.SS. Al-khilkhali H.JB. Antimicrobial susceptibility patterns of Klebsiella pneumoniae isolated from older smokers and non-smokers of inpatients in intensive care unit infected with chronic pneumonia in AL-Najaf hospital, Iraq. /J. Pharm. Sci. & Res. Vol. 10(5), 2018, 1093-1097.

Anoar , K. A., Ali, F. A. & Omer , S. A. Detection of Mettalo [Beta]-Lactamase enzyme in some Gram Negative bacterial isolates from burn patients in Sulaimani city /Iraq European Scientific Journal. 2014; 10. (3).

Aziz, R. A. R. & AL-JUBORI, S. S. Genetic Elements Responsible for extreme drug resistance (XDR) in *Klebsiella Pneumoniae* var Pneumoniae isolated from clinical samples of Iraqi patients. World Journal of Pharmaceutical Research. 2016; ( 5) : 2.

Bahar, I. H. Molecular characterization of TEM-and SHV-derived extended-spectrum beta-lactamases in hospital-based Enterobacteriaceae in Turkey. 2005; 58, 162-167.

Batarseh H, A., Soneah , S., Mardeni , R., Elmandi , K. & Ashour , N. A. Antibiotic resistant pattern of multi drug resistant and extended spectrum beta lactmase producing E.coli urinary isolates of Quine Rania Al- Abdullah hospital children. Zagazig University Medical Journal. 2015; 19.N.5.

Bauer AW, Kirby WM, Sherris JC, Turck M. Antibiotic susceptibility testing by a standardized single disk method. American Journal of Clinical Pathology. 1966;45(4):493-6.

Bora, A., Hazarika , N. K., Shukla , S. K., Prasad , K. N., Sarma , J. B. & Ahmed , G. Prevalence of blaTEM, blaSHV and blaCTX-M genes in clinical isolates of *Escherichia coli* and *Klebsiella Pneumoniae* from Northeast India. Indian journal of Pathology and Microbiology. 2014; 57, 249.

Catalan-Najera, J. C., Garza-Ramos, U. & Barrios-Camacho, H. 2017. Hypervirulence and hypermucoviscosity: Two different but complementary Klebsiella spp. phenotypes? Virulence, 8, 1111.

CLSI 2018. Performance standards for antimicrobial susceptibility testing. Wayne, PA: Clinical and Laboratory Standards Institute.

Diestra , K., Coque , T. M., Miro , E., Oteo , J., Nicolau , C. J., Campos , J., Moya , B., Curiao , T., Perez-Vazquez , M. & Canton , R. Characterization and molecular epidemiology of ESBL in *Escherichia coli* and *Klebsiella Pneumoniae* in 11 Spanish hospitals (2004). Enfermedades infecciosas y microbiologia clinica. 2008; 26, 404-410.

Doosti , A., Pourabbas , M., Arshi , A., Chehelgerdi , M. & Kabiri , H. TEM and SHV genes in Klebsiella pneumoniae isolated from cockroaches and their antimicrobial resistance pattern. Osong public health and research perspectives. 2015; 6, 3-8.

Elnagar, W., Habib, E., Shokralla, S., Barwa, R., Abdel Galil , K. & Abdulmageed , E. Phenotypic and genotypic characterization of Extended Spectrum Beta-lactamases produced by Enterobacteriaceae.The Egyptian J. Med. Microbiol.2010. 19, 59-66.

Garrec, H., Drieux-Rouzet, L., Golmard, J.-L., Jariler, V. & Robert, J. Comparison of nine phenotypic methods for detection of extended-spectrum β-lactamase production by Enterobacteriaceae. Journal of clinical microbiology.2011; 49, 1048-1057.

Guo, X., Cao, Z., Dai, Z., Li, Y., He, X., Hu, X., Tian, F. & Ren, Y. Antimicrobial Susceptibility and Molecular Epidemiology of Multidrug-Resistant Klebsiella pneumoniae in Central China. Jpn J Infect Dis. 2017; 70, 229-234.

Gupta, A., Ampofo, K., Rubenstein, D. & Saiman, L. Extended spectrum β lactamase-producing Klebsiella pneumoniae infections: a review of the literature. Journal of perinatology. 2003. 23, 439.

Haeggman , S., Lofdahl , S., Paauw , A., Verhef , J. & Brisse , S. Diversity and evolution of the class A chromosomal beta-lactamase gene in *Klebsiella Pneumoniae*. Antimicrobial agents and chemotherapy. 2004; 48, 2400-2408.

Hagia , H., Watanabe , N., Maki , M., Murase , T. & Otsuka , F. Clinical utility of string test as a screening method for hypermucoviscosity-phenotype Klebsiella pneumoniae. Acute medicine & surgery. 2014; 1, 245-246.

Hassan , M. I., Alkharsah , K. R., Alzahrani , A. J., Obeid , O. E., Khamis , A. H. & Diab, A. Detection of extended spectrum beta-lactamases-producing isolates and effect of AmpC overlapping. The Journal of Infection in Developing Countries. 2013; 7, 618-629.

Hawser , S. P., Bouchillon , S. K., Lascols , C., Hackel , M., Hoban , D. J., Badal , R. E., Woodford , N. & Livermore , D. M. Susceptibility of *Klebsiella Pneumoniae* isolates from intra-abdominal infections and molecular characterization of ertapenem-resistant isolates. Antimicrobial agents and chemotherapy. 2011; 55, 3917-3921.

Iliyasu , M., Uba, A. & Agbo, E. Phenotypic detection of multidrug resistant extended-spectrum beta-lactamase (ESBL) producing Escherichia coli from clinical samples. African Journal of Cellular Pathology.2018; 9, 25-32.

Irajian , G., Jazayeri, M. A. & Beheshti , A. Prevalence of extended-spectrum beta lactamase positive and multidrug resistance pattern of Escherichia coli and *Klebsiella Pneumoniae* isolates, Semnan, Iran. Iranian Journal of Pathology. 2009; 4 (3), 128-132.

Jean Ean, S.-S. & Hsueh , P.-R. High burden of antimicrobial resistance in Asia. International journal of antimicrobial agents. 2011. 37, 291-295.

Kazemian , H., Heidari , H., Ghanavati , R., Ghafourian , S., Yazdani , F., Sadeghifard , N., Valadebigi , H., Maleki , A. & Pakazad , I. Phenotypic and Genotypic characterization of ESBLs, AmpC and Carbapenemase-Producing *Klebsiella Pneumoniae* and Escherichia coli Isolates. Med Princ Pract. 2019 ; 28, 547–551.

Khalid , H. M., Yosif , S. Y. & Jubarel , J. M. 2013. Bacteriological and Molecular Characterization of Extended Spectrum Β-Lactamases in Clinical Isolates of *Klebsiella Pneumoniae* Isolated From Kurdistan Region, Iraq. Science Journal of University of Zakho. 2013; 1, 158-163.

Khosravi , A. D., Hoveizavi , H. & Mehdinejad , M. Prevalence of *Klebsiella Pneumoniae* encoding genes for CTX-M-1, TEM-1 and SHV-1 extended-spectrum beta lactamases (ESBL) enzymes in clinical specimens. Jundishapur Journal of Microbiology. 2013; 6.

Latipour M., Gholipour , A. & Damavandi , M. S. Prevalence of extended-spectrum beta-lactamase-producing *Klebsiella pneumoniae* isolates in nosocomial and community-acquired urinary tract infections. Jundishapur journal of microbiology. 2016. 12;9(3):e31179.

Lee, C.-R., Lee, J. H., ParkK, K. S., Kim, Y. B., Jeong, B. C. & Lee, S. H. Global dissemination of carbapenemase-producing Klebsiella pneumoniae: epidemiology, genetic context, treatment options, and detection methods. Frontiers in microbiology.2016. 7, 895.

Luo, Y., Wang, Y., YeE, L. & Yang, J. Molecular epidemiology and virulence factors of pyogenic liver abscess causing Klebsiella pneumoniae in China. Clinical Microbiology and Infection.2014. 20, O818-O824.

Malik , T., Naim , A. & Saeed , A. Molecular Detection of TEM, SHV and CTX-M Genes Among Gram-negative Klebsiella Isolates. Current drug delivery 2018; 15, 417-423.

Mawlood A. H., Omer , S. A., Jalal , S. T. & Haji , S. H. Molecular detection of SHV-Type ESBL in E.coli and K. pneumoniae and their antimicrobial resistance profile. Zanco Journal of Medical Sciences, .2018.22, 262-272.

[Mohammed](https://www.ncbi.nlm.nih.gov/pubmed/?term=Mohammed%20I%5BAuthor%5D&cauthor=true&cauthor_uid=31372151) I. and Abass E. Phenotypic detection of Extended Spectrum β-Lactamases (ESBL) among gram negative uropathogens reveals highly susceptibility to imipenem. [Pak J Med Sci](https://www.ncbi.nlm.nih.gov/pmc/articles/PMC6659091/). 2019. 35(4): 1104–1109.

Moland , E. S., Kim , S.-Y., Hong , S. G. & Thomson , K. S.Newer β-Lactamases: clinical and laboratory implications, Part I. Clinical Microbiology Newsletter. 2008. 30, 71-77.

Navon-Venzea , S., Kondratyeva ,K. & Carattoli , A. *Klebsiella Pneumoniae*: a major worldwide source and shuttle for antibiotic resistance. FEMS microbiology reviews. 2017. 41, 252-275.

Paterson , D. L. & Bonomo , R. A. Extended-Spectrum β-Lactamases: a Clinical Update. Clinical Microbiology Review . 2005. 18(4): 657-686.

Philippon , A., Slama , P., Deny , P. & Labia , R. A structure-based classification of class A β-lactamases, a broadly diverse family of enzymes. Clinical microbiology reviews.2016; 29, 29-57.

Pishtiwan , A. H. & Khadija , K. M. Prevalence of blaTEM, blaSHV, and blaCTX-M Genes among ESBL-Producing Klebsiella pneumoniae and Escherichia coli Isolated from Thalassemia Patients in Erbil, Iraq. Mediterranean journal of hematology and infectious diseases. 2019. 11(1):e2019041.

Pitout , J. D. & Laupland , K. B. Extended-spectrum β-lactamase-producing Enterobacteriaceae: an emerging public-health concern. The Lancet infectious diseases. 2008 ; 8, 159-166.

Qader , M. K. Bacteriological and Molecular Study Characterization of Klebsiella pneumoniae Isolated from Different Clinical Specimens. UKH Journal of Science and Engineering. 2019; 3, 22-30.

Rangachari , R. K., Kumar, M. S. & Priyadarsini, I. Detection of extended spectrum beta-lactamase producing gram negative bacilli in urinary isolates. Int J Biol Med Res. 2010; 1, 130-132.

Ranjbar , R., Fatahian K. Kelishadrokhi , A. & Chehlgerdi , M. Molecular characterization, serotypes and phenotypic and genotypic evaluation of antibiotic resistance of the Klebsiella pneumoniae strains isolated from different types of hospital-acquired infections. Infect Drug Resist. 2019; 12, 603-611.

Remya , P., Shanthi , M. & SEKAR, U. 2019. Characterization of virulence genes associated with pathogenicity in *Klebsiella Pneumoniae*. Indian journal of medical microbiology. 2019; 37, 210.

Rozwandwicz , M., Brouwer , M., Fischer , J., Wagnaar , J., Gonzalez –zorn , B., Guerra , B., Mwvius , D. & Hordijk , J. Plasmids carrying antimicrobial resistance genes in Enterobacteriaceae. J Antimicrob Chemother.2018.1;73(5):1121-1137.

Rudresh , S. & Nagrathnamma , T. Extended spectrum β-lactamase producing Enterobacteriaceae & antibiotic co-resistance. The Indian journal of medical research. 2011; 133, 116.

Schwarz , S., Silley , P., Simjjee , S., Woodford , N., Van Duijkeren , E., Johnson , A. P. & Gaastra , W. 2010. Assessing the antimicrobial susceptibility of bacteria obtained from animals. Journal of antimicrobial chemotherapy. 2010. 65, 601-604.

Shariif , N. M., Sreedevi , B., Chaitanya, R. & Sriltha , C. Detection of extended spectrum beta-lactam (ESBL) resistance in Pseudomonas species of canine origin. The Pharma Innovation.2017. 6, 89.

Shon , A. S., Bajiwa , R. P. & Russo, T. A. Hypervirulent (hypermucoviscous) Klebsiella pneumoniae: a new and dangerous breed. Virulence. 2013; 4, 107-118.

Tawgozy , F. H. A. & Amin , B. K. Molecular Analysis of Klebsiella Pneumoniae Isolated from UTI Patients in Erbil City. ZANCO Journal of Pure and Applied Sciences. 2018; 30, 1-9.

Tsai , S.-S., Huang , J.-C., Chean , S.-T., Sun, J.-H., Wang G, C.-C., Lin, S.-F., Hsu, B., Lin, J.-D., Hunga, S.-Y. & Hunga , Y.-Y. Characteristics of Klebsiella pneumoniae bacteremia in community-acquired and nosocomial infections in diabetic patients. Chang Gung Med J.2010. 33, 532-9.

Woodford , N., Turton , J. F. & Livermoor , D. M. Multiresistant Gram-negative bacteria: the role of high-risk clones in the dissemination of antibiotic resistance. FEMS microbiology reviews. 2011; 35, 736-755.
