# Supplementary material for: Phenotypic and genotypic detection of extended spectrum beta lactamase enzyme in Klebsiella pneumoniae
Source: PLoS One. 2022 Sep 29;17(9):e0267221. doi: 10.1371/journal.pone.0267221 (PMC9522270; doi:10.1371/journal.pone.0267221)
Supplement: Attachment — Submitted filename: renamed_36ab1.docx [file pone.0267221.s003.docx]

| N0 | **Line number** | **Reviewer’s Comments/Suggestions** |
| --- | --- | --- |
| 1 | Abstract | Most results of antibiotic not clarify clearly in abstract section |
|  | Keywords | Need to arrange alphbetically |
| 2 |  | The manuscript contains many grammatical errors that must be corrected especialy the discussion and introduction section need too much correction |
| 3 | highlighted in Green | Correct the word |
| 4 | highlighted in Red | Delete word or character |
| 5 |  | Many Word not writed in fully form like” EBLS,ICU,U.S.A,MICs,OPD)and others, also the term EBLS sometimes writed “ESBLS” other palces writed “ESBLs” it must write in unique and scinetific style |
| 6 | Highlighted in Yellow | Give a space |
| 7 | Results | - İn this sectio the concentration of each antibiotiv must mentioned inside the (table 4).  - (P-value with 0.00001) this wrong value (correct the value). |
| 8 | Discussion | The authors mentioned in this section this wrong reference lined with yellow” The other drug such as amoxicillin-clavulanic acid also shows high resistance rate (96.29%), the same result was reported in Erbil , Najaf and Duhok / Iraq (Gupta etal.(2003), (Aljanaby etal.(2018), (Qadir (2019). Respectively. So Gupta and his collegous study founded in U.S.A |
| 9 | References | All the refernce not in one unique style; the year of publication sometime after the name of authors and sometimes after the title of article, sometiems after the year of publication come comma others semicolon |
| 10 | Others important notice | - Many word wtrited wrongly like „standardise,Fifty four,30 µg must become  30µg (without any space),TEM and SHV writed with different format like balSHV,blasCTM  - Table 1,2 need to re-type in word format not in picture foramt  - Chi-sequare and correlation analysis test mentioned in statistical anlalysis section but actualy not calculate for the data (for comparision), the authors must delete these characters , also the authors must mentioned which version of M.soft excel they used  - Table 3, 4,5 ,6 and 7 must re-type again with word format adn without coluration .  - The manuscript not contanin conclusion and any Acknowledgement. |
