# Supplementary material for: Phenotypic and genotypic detection of extended spectrum beta lactamase enzyme in Klebsiella pneumoniae
Source: PLoS One. 2022 Sep 29;17(9):e0267221. doi: 10.1371/journal.pone.0267221 (PMC9522270; doi:10.1371/journal.pone.0267221)
Supplement: Attachment — Submitted filename: Respons to reviewer.docx [file pone.0267221.s004.docx]

1/ all Klebsiella pneumoniae were checked.

2/ tables written and putted in word format

3/ figure uploaded in APACE style

4/ Reference checked according to submission guide line

5/ all reviewer comment checked step by step

6/ 2 file uploaded one is manuscript ( re new one after correction ) , other revised one as follow : green word : corrected and newly added according to reviewer comment , red words : deleted word

7/ cover page checked according to file template of plos one \
